# Examining Sources of Error in PCR by Single-Molecule Sequencing

**DOI:** 10.1371/journal.pone.0169774

**Published:** 2017-01-06

**Authors:** Vladimir Potapov, Jennifer L. Ong

**Affiliations:** New England Biolabs, Ipswich, Massachusetts, United States of America; University of Helsinki, FINLAND

## Abstract

Next-generation sequencing technology has enabled the detection of rare genetic or somatic mutations and contributed to our understanding of disease progression and evolution. However, many next-generation sequencing technologies first rely on DNA amplification, via the Polymerase Chain Reaction (PCR), as part of sample preparation workflows. Mistakes made during PCR appear in sequencing data and contribute to false mutations that can ultimately confound genetic analysis. In this report, a single-molecule sequencing assay was used to comprehensively catalog the different types of errors introduced during PCR, including polymerase misincorporation, structure-induced template-switching, PCR-mediated recombination and DNA damage. In addition to well-characterized polymerase base substitution errors, other sources of error were found to be equally prevalent. PCR-mediated recombination by *Taq* polymerase was observed at the single-molecule level, and surprisingly found to occur as frequently as polymerase base substitution errors, suggesting it may be an underappreciated source of error for multiplex amplification reactions. Inverted repeat structural elements in *lacZ* caused polymerase template-switching between the top and bottom strands during replication and the frequency of these events were measured for different polymerases. For very accurate polymerases, DNA damage introduced during temperature cycling, and not polymerase base substitution errors, appeared to be the major contributor toward mutations occurring in amplification products. In total, we analyzed PCR products at the single-molecule level and present here a more complete picture of the types of mistakes that occur during DNA amplification.

## Introduction

Genetic variation underlies many fundamental aspects of biology. Mutations drive speciation or cause disease, and their detection has been critical to our understanding of evolution and translational medicine. During our lifetime, spontaneous mutations accumulate in somatic cells, and much progress has been made in understanding their contribution to cancer and aging [1]. Understanding disease progression and ultimately, optimizing therapy, often requires the detection of rare mutations in heterogeneous samples. For example, in cases where early somatic mutation in tissue leads to a mixed population of cancer and normal cells, earlier detection of the low-abundance mutations for tumor indication could lead to earlier diagnosis and treatment. Recent innovations in next-generation sequencing (NGS) technologies have enabled the detection of novel mutations, yet, detecting low frequency variation among a mixed population remains challenging due to low sample input and amplification bias. Prior to sequencing, many sample preparation workflows employ DNA amplification, particularly the Polymerase Chain Reaction (PCR). Consequently, errors that arise during PCR lead to false positive mutations and can obscure sequencing results, making it especially challenging to identify rare genetic variation [2]. In addition, the inherently high error rate of next-generation sequencing technologies requires additional steps to distinguish true mutation events from sequencing errors and is an active area of research [3–6].

In an effort to understand PCR errors, much attention has focused on DNA polymerase base substitution errors. DNA polymerase replication fidelity has been extensively studied with multiple methods and various assay conditions. Assays to determine replication fidelity have utilized various approaches: blue/white screening ([7], [8], [9], and reviewed in [10]), forward mutation [11], denaturing gradient gel electrophoresis [12, 13], high throughput Sanger sequencing [14], or next-generation sequencing [5, 15, 16]. Differences in assay methodology, reaction conditions, template sequences and error reporting units can yield different absolute values for error rates. For example, the reported error rate for *Taq* DNA polymerase I, can range over 10-fold, from 1 × 10^−5^ to 2 × 10^−4^ errors/base/doubling [9, 13]. Error rates are traditionally reported as errors per base per doubling event, which normalizes the raw error rate (the fraction of observed errors after sequencing a PCR product) to the number of doubling events that occur during amplification. Normalizing raw error rates to the number of template doublings corrects for the propagation of errors during exponential amplification and the different replication efficiencies of different polymerases. However, more recent fidelity studies utilizing next-generation sequencing typically report error rates as error per base per number of PCR cycles [5, 15, 16]. As DNA replication per PCR cycle is not perfectly efficient, the number of doubling events is less than the number of PCR cycles, making it more difficult to compare studies with different error reporting units. In addition, reaction conditions, particularly dNTP and magnesium concentration, have a large influence on fidelity, with the range of low to high fidelity results spanning 3 orders of magnitude for *Taq* DNA polymerase I, from 1/3,200 to 1/300,000 errors/base [8]. To compensate for assay variation and reaction conditions, polymerase error rates are often reported relative to a reference polymerase, typically *Taq* DNA polymerase.

For detecting polymerase misincorporation, direct sequencing of amplification products, either through high-throughput dideoxy sequencing or next-generation sequencing, yields direct information regarding the frequency and types of errors introduced during replication. Sanger sequencing generates limited data sets, and is more suitable for measuring the fidelity of low-fidelity polymerases without proofreading 3’-5’ exonuclease activity. Enough Sanger sequencing data can be easily generated to accurately measure the fidelity of *Taq* DNA polymerase I, which makes a mistake once every tens of thousands of bases replicated. Measuring the replication fidelity for high fidelity polymerases with a proofreading 3’-5’ exonuclease domain (such as the archaeal Family B polymerases) requires sequencing a larger number of bases to count a statistically significant number of errors. In this case, next-generation sequencing methods are more suitable. Next-generation sequencing platforms provide vast sequencing data (on the order of millions to billions of bases read), but also generate inherent sequencing errors that are typically orders of magnitude higher than the errors introduced during amplification [3, 5]. To distinguish between sequencing errors and true polymerase errors, Kinde *et al*. used Illumina sequencing to detect rare mutation events and measure polymerase error rates [5, 17]. Lee *et al*. also used a similar approach to measure the fidelity of DNA polymerases performing a single round of DNA synthesis [16]. In both methods, individual molecules were tagged with a unique molecular index prior to Illumina sequencing. Amplification and redundant sequencing of each index distinguished sequencing errors from true polymerase errors. Amplification of molecular indexes has its own challenges [17]. Further studies indicated that the background error rate of this method is approximately 2.5 × 10^−6^ errors/base based on the integrity of the molecular index [3, 17]. The error rates of very accurate polymerases also fall in this range, thus highlighting the challenge of measuring their fidelities [5, 15, 16].

Pacific Biosciences Single Molecule Real-Time (SMRT) sequencing technology has also been utilized to measure replication fidelity during PCR and has unique advantages for determining replication error rates. PCR products can be directly sequenced, without molecular indexing or an intermediary amplification step (as needed for Illumina sequencing). In SMRT sequencing, accuracy is achieved by sequencing the same molecule multiple times and deriving a highly accurate consensus sequence for each read, which can be used to identify true replication errors. Using PacBio SMRT sequencing, Hestand *et al*. measured the base substitution error rates of several PCR enzymes [15]. Single-molecule sequencing of individual amplification products by next-generation sequencing has made it easier to assay polymerase fidelity, but previous studies were limited to examining polymerase base substitution errors.

In addition to base substitution errors, other larger-scale errors have been observed in PCR products. Chimeric amplification products, generated by polymerase template-switching (recombination) on closely-related templates, have been observed and cause problems with interpreting assay results. For example, replication chimeras are known to cause species misidentification in 16S ribosomal sequencing for microbe identification [18]. Similarly, chimeric PCR products were also observed after amplification and sequencing of human leukocyte antigen (HLA) genes for genotyping and lead to incorrect genotype assignment [19]. Reports of the prevalence of PCR recombination vary, but it has been estimated that up to 40% of PCR products amplified from mixed populations are artificial chimeras [20, 21]. Recombinants can arise when partially extended primers anneal to partially homologous target sequences and are extended at a later PCR cycle. Studies have also shown that recombinants can also arise between complementary strands, or even during a single round of polymerase extension [22]. The rate of PCR-mediated recombination by polymerases has been studied by a phenotypic assay and next-generation sequencing, but has yet to be directly assayed by single-molecule sequencing methods [2, 23, 24].

In this report, we utilized a single-molecule sequencing assay to accurately and directly sequence PCR products to capture the various types of errors generated during PCR: base substitution errors, template-switching, PCR-mediated recombination and non-enzymatic DNA damage. The base substitution error rates were measured for several wild-type and engineered DNA polymerases, and found that for extremely accurate polymerases, such as Q5 DNA polymerase, polymerase substitution errors are not the major contributor to base substitution errors in amplification products. Thermocycling-induced DNA damage contributed a base substitution error rate that is more than two-fold higher than the base substitution rate of Q5 DNA polymerase. Furthermore, deep sequencing of a *lacZ* gene fragment revealed multiple structured regions susceptible to polymerase template-switching events, which are detected as inversion events in individual sequencing reads. In addition, PCR-mediated recombination by *Taq* polymerase was observed at the single-molecule level, and found it to occur as frequently as polymerase misincorporation. As PCR is utilized upstream of next-generation sequencing technologies for target enrichment or as part of the library preparation workflow, understanding potential error pathways is critical for sequencing accuracy and genetic analysis.

## Results

### Identifying polymerase base substitution errors in PCR

SMRT sequencing was first used to measure the base substitution error rates of DNA polymerases. To validate the use of single-molecule sequencing for measuring replication fidelity, the fidelity of *Taq* polymerase was measured using both PacBio SMRT and traditional Sanger sequencing. For the same LacZ-1 sample, the results were similar: PacBio sequencing produced an error rate of 1.8 × 10^−4^ errors/base/doubling and Sanger sequencing produced an error rate of 1.3 × 10^−4^ errors/base/doubling (Table 1). However, sequencing 576 colonies by Sanger sequencing produced 323,802 double-pass sequenced bases, whereas 1 SMRT Cell produced 35,879,784 high-quality consensus bases; over two orders of magnitude more data. The Sanger data set indicated that most errors introduced by *Taq* polymerase were base substitutions (98.8%) with a few deletions (1.2%) and no insertions detected. PacBio sequencing gave a similar distribution for base substitutions (97.3%), deletions (2.6%) and insertions (0.1%), although the fraction of insertions and deletions was slightly higher than those observed by Sanger sequencing. The mutational spectrum was similar for both sequencing methods as well; A→G/T→C transitions were identified as the major class of substitution errors for *Taq* polymerase (66% by both methods), followed by G→A/C→T (21 vs 19%), and A→T/T→A (10% vs 9.3%) (Table 2).

**Table 1:** Error rate of *Taq* DNA polymerase.

Amplicon	Substitution rate	Deletion rate	Insertion rate	Total error rate	Total bases
***Sanger (dideoxy)***					
LacZ-1	1.2 × 10^−4^ (98.8%)	1.6 × 10^−6^ (1.2%)	- (0.0%)	1.3 × 10^−4^	323,802
***Pacific Biosciences RSII***					
LacZ-1	1.7 × 10^−4^ (97.3%)	4.7 × 10^−6^ (2.6%)	1.8 × 10^−7^ (0.1%)	1.8 × 10^−4^	35,879,784
LacZ-2	1.7 × 10^−4^ (96.1%)	5.1 × 10^−6^ (2.9%)	1.8 × 10^−6^ (1.0%)	1.8 × 10^−4^	15,857,446
DNA-1	1.4 × 10^−4^ (97.2%)	3.9 × 10^−6^ (2.8%)	1.2 × 10^−7^ (0.1%)	1.4 × 10^−4^	18,680,811
DNA-2	1.4 × 10^−4^ (97.5%)	3.4 × 10^−6^ (2.4%)	1.5 × 10^−7^ (0.1%)	1.4 × 10^−4^	27,978,748

Reported error rates are per base per doubling as detailed in Materials and Methods. Numbers in parentheses are percentages of the total error rate.

**Table 2:** Distribution of individual error types for *Taq* DNA polymerase.

Sequencing method	A→G, T→C (%)	G→A, C→T (%)	A→T, T→A (%)	A→C, T→G (%)	G→C, C→G (%)	G→T, C→A (%)
Sanger (dideoxy)	66	21	10	0.9	1.4	0.9
Pacific Biosciences RSII	66	19	9.3	2.0	1.6	2.0

Data derived from sequencing LacZ-1 amplicon.

**Fig 1:**
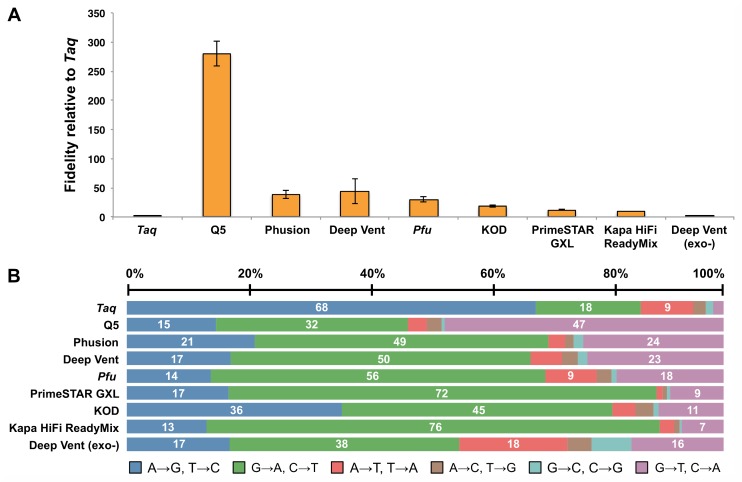
Fidelity measurements and mutational spectrum of DNA polymerases. (A) Base substitution error rates of various DNA polymerases relative to Taq polymerase. (B) Proportion of each type of base substitution error as a percentage of the total errors for each polymerase.

The base substitution error rates for a wider range of wild-type and engineered DNA polymerases were determined by single-molecule sequencing, and the average values for 3 different amplicons are reported in Table 3 and Fig 1 (for additional details see S1 Table). Only base substitution errors were reported, since these are the predominant class of polymerase errors, as reported here and elsewhere (15). The base substitution rates for the DNA polymerases assayed spanned 3 orders of magnitude with the highest fidelity observed for Q5 DNA polymerase (5.3 × 10^−7^ sub/base/doubling) and the lowest for exonuclease-deficient Deep Vent (exo-) polymerase (5.0 × 10^−4^ sub/base/doubling) and the inherently non-proofreading *Taq* polymerase (1.5 × 10^−4^ sub/base/doubling) (Table 3). The contribution of proofreading activity to fidelity was over 2 orders of magnitude for Deep Vent DNA polymerase: comparing the error rate of the exonuclease-proficient to the exonuclease-deficient polymerase increased the error rate by 125-fold, from 4.0 × 10^−6^ to 5.0 × 10^−4^ sub/base/doubling. Other wild-type and engineered high fidelity DNA polymerases had a range of base substitution error rates: Phusion (3.9 × 10^−6^ sub/base/doubling), *Pfu* (5.1 × 10^−6^ sub/base/doubling), PrimeSTAR GXL (8.4 × 10^−6^ sub/base/doubling), KOD (1.2 × 10^−5^ sub/base/doubling) and Kapa HiFi HotStart ReadyMix (1.6 × 10^−5^ sub/base/doubling).

**Table 3:** Substitution error rates measured by PacBio single-molecule sequencing.

DNA Polymerase	Substitution rate ^a^	Accuracy ^b^	Fidelity, rel. to *Taq* ^c^	Total bases
*Taq*	1.5 × 10^−4^ (± 0.2 × 10^−4^)	6,456	1	98,396,789
Q5	5.3 × 10^−7^ (± 0.9 × 10^−7^)	1,870,763	280	112,619,228
Phusion	3.9 × 10^−6^ (± 0.7 × 10^−6^)	255,118	39	118,262,939
Deep Vent	4.0 × 10^−6^ (± 2.0 × 10^−6^)	251,129	44	106,217,940
*Pfu*	5.1 × 10^−6^ (± 1.1 × 10^−6^)	195,275	30	79,614,976
PrimeSTAR GXL	8.4 × 10^−6^ (± 1.1 × 10^−6^)	118,467	18	118,964,566
KOD	1.2 × 10^−5^ (± 0.2 × 10^−5^)	82,303	12	121,234,438
Kapa HiFi HotStart ReadyMix	1.6 × 10^−5^ (± 0.3 × 10^−5^)	63,323	9.4	101,742,963
Deep Vent (exo-)	5.0 × 10^−4^ (± 0.1 × 10^−4^)	2,020	0.3	60,218,605

^a^ Reported error rates are per base per doubling as detailed in Materials and Methods. Standard deviations were determined based on sequencing several samples and are given here in brackets.

^b^ Accuracy is calculated as 1 over substitution rate such that accuracy is a number of bases over which 1 substitution error is expected.

^c^ Fidelity relative to *Taq* numbers are computed separately for each amplicon (LacZ-1, LacZ-2, DNA-1, DNA-2) and the average number is reported per DNA polymerase. Individual values are available in S1 Table.

The mutational spectrum differed significantly for Family A and Family B DNA polymerases (Fig 1B and S2 Table). As measured by SMRT sequencing, for the Family A *Taq* polymerase, the majority of base substitutions were A→G/T→C transitions (68%) and G→A/C→T (18%), with fewer transversions A→T/T→A (8.8%) (S2 Table). Therefore, the majority of base substitution events for Family A DNA polymerase appeared to occur at A:T pairs, and was consistent with other unpublished results (J.L.O and V.P.) and previous reports [14]. Proofreading archaeal Family B polymerases had a different mutational spectrum: the majority of base substitution errors made by Deep Vent polymerase were G→A/C→T transitions (50%), with fewer A→G/T→C transitions (17%) and G→T/C→A (23%), low A→T/T→A (5.2%) and almost no A→C/T→G and G→C/C→G transversions were observed. A similar distribution of base substitutions was observed for other proofreading polymerases tested: Q5, Phusion, *Pfu*, KOD, PrimeSTAR GXL and Kapa HiFi DNA polymerases. Inactivating the editing function for Deep Vent DNA polymerase also changed the mutational spectrum: the largest class of mutations remained G→A/C→T transitions (50% and 38%), and A→T/T→A transversions increased from 5.2 to 18% for the exonuclease-deficient polymerase, indicating that the polymerase and exonuclease domains may have their own distinct substrate specificities.

### Evidence of template-switching at cruciform DNA structures in *lacZ*

**Fig 2:**
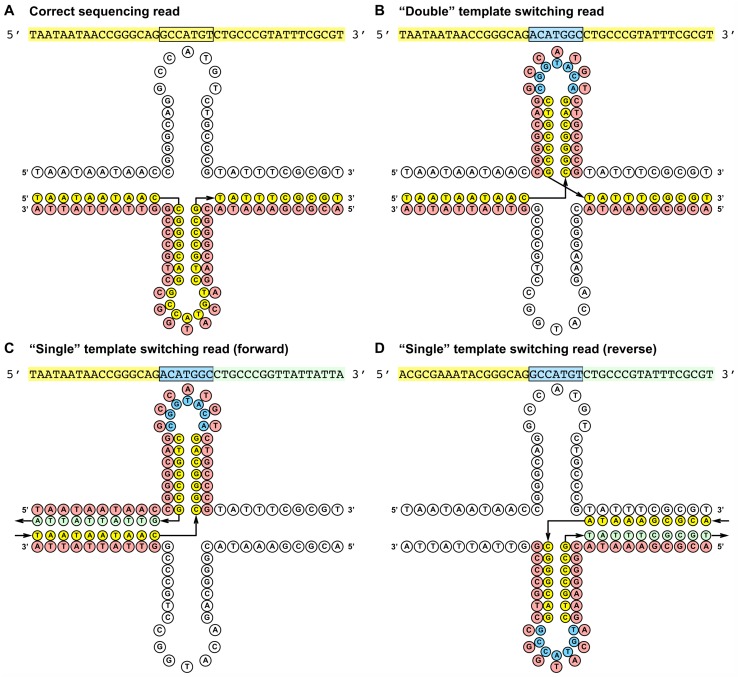
Schematic of template-switching in *lacZ*. Replication of potential cruciform structures at *lacZ* coordinates 3083..3103 without template-switching (A) and with template-switching (B, C and D) between top and bottom strands. Three classes of template-switching events were identified based on sequence context: double-switching events (B) and single-switch events on the bottom (C) or top (D) strands. Similar sites were identified at locations 185..222 and 1843..1981 in *lacZ*.

For most of the polymerases assayed, base substitution rates were elevated for the *lacZ* amplicon compared to the other artificial genes DNA-1 and DNA-2 (S1 Table). Closer inspection revealed mutational hotspots in *lacZ* located in regions containing inverted repeats capable of forming cruciform structures (Fig 2). The “errors” detected by comparison to the reference sequence did not appear to be a series of single base substitution errors, but were instead larger-scale inversion events. The inverted sequence corresponded exactly to replication of the loop region of the hairpin on the opposite strand, and was consistent with polymerase template-switching between strands (Fig 2). In total, three regions of *lacZ* were identified that produced evidence of template-switching inversions and all were flanked by inverted repeats (Table 4). The frequency of template-switching at Inversion 2 and 3 was measured by the presence of sequence inversions in unfiltered sequencing reads from the LacZ-2 amplicon for all assayed polymerases (Table 5). An earlier study identified Inversion 1 in *lacZ* that produced template-switching events, and the frequency of template-switching at this site was not measured in this study (J.L.O and V.P., unpublished data). By analyzing the sequence context of the inversion events, sequencing reads were observed that corresponded to both single- and double- template-switching events as illustrated in Fig 2. The percentage of total reads inversions were summarized in Table 5. Deep Vent, *Pfu* and KOD DNA polymerases were particularly susceptible to template-switching, with up to 1.54%, 0.42% and 0.40% of all reads, respectively, containing the distinguishing sequence inversion at either region. For Deep Vent DNA polymerase, the majority of template-switching events observed were from single-switch events on the reverse strand (1.35% of all reads, S3 Table). Single switch events on the forward strand were not as frequently observed (0.006% of reads), because the size of the replication product (227 nt) was too small to be sequenced by MagBead loading on the PacBio RSII sequencer [14]. Sequencing reads with double template-switching events were also observed at both inverted regions (0.18 or 0.16% of reads for Deep Vent polymerase, S3 Table). Interestingly, the exonuclease-deficient variant of Deep Vent polymerase had a much lower total frequency of template-switching at the two inverted regions (0.03 and 0.01% of reads; Table 5), suggesting that abolishing proofreading activity may increase the ability of the polymerase to accurately replicate through these structured elements.

**Table 4:** Regions in *lacZ* with observed template-switching events.

Inversion	Location in *lacZ* gene	Location in LacZ-2 amplicon ^a^	Stem length	Loop length
Inversion 1	185..222	n.a.	16	6
Inversion 2	1843..1981	45..183	10	129
Inversion 3	3083..3103	1285..1305	8	5

^a^ The length of LacZ-2 amplicon in 1353 nt. Inversion 2 is located close to the 5’ end of the amplicon and Inversion 3 is close to the 3’ end. Inversion 1 is not covered by LacZ-2 amplicon.

**Table 5:** Percentage of strands with inversions.

Enzyme	Location in *lacZ* gene	
	1843..1981	3083..3103
*Taq*	n.d. ^a^	n.d.
Q5	0.003%	0.001%
Phusion	n.d.	0.02%
Deep Vent	1.54%	0.17%
*Pfu*	0.42%	0.003%
PrimeSTAR GXL	n.d.	0.004%
KOD	0.01%	0.40%
Kapa HiFi HotStart ReadyMix	0.006%	0.003%
Deep Vent (exo-)	0.03%	0.01%

^a^ “n.d.” indicates that template-switching reads were not detected.

### PCR-mediated recombination

**Fig 3:**
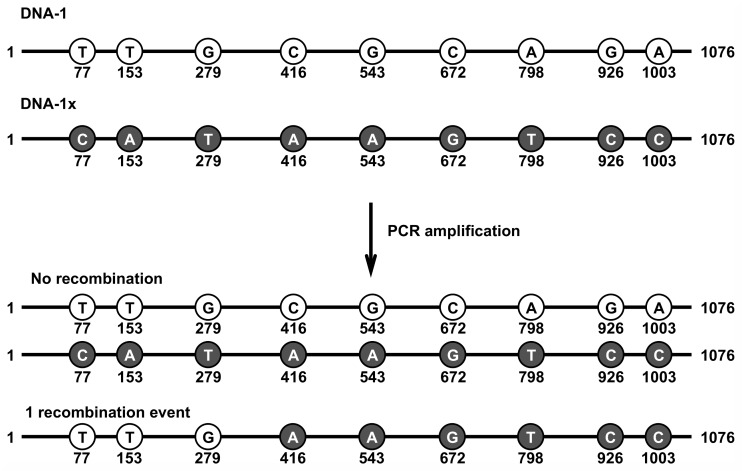
Schematic of measuring PCR-mediated recombination rate. Two template pairs, differing by single point mutations (markers) spaced at regular intervals across the gene are co-amplified. Recombination products contain markers from both templates.

Intrigued by the observation of template-switching across structured elements, an assay was developed to measure template-switching between different DNA molecules, frequently described as PCR-mediated recombination [25]. During amplification of a mixed population of related sequences, recombination is thought to occur when a partially extended primer from one template anneals to a different (but closely related) template molecule in a later round of PCR, where further extension results in a chimeric product. A single-molecule sequencing assay was developed to mimic amplification of closely related sequences, using pairs of artificial genes that differ by point mutations spaced at regular intervals across the gene. After amplification of the mixed templates, recombination events can be detected by SMRT sequencing when a sequencing read has markers from both templates (Fig 3). Since recombination can only be detected between two different templates (recombination between identical templates is cryptic), the total number of recombination events is assumed to be double the amount detected by sequencing. The total number of recombination events, and the resulting recombination rate per base per doubling event, was determined for *Taq* polymerase (Table 6). Recombination occurs at an average rate of 1.1 × 10^−4^ base/doubling for *Taq* DNA polymerase for the two template pairs, after normalization for error propagation in PCR. The locations of recombination events were mapped along the reference genes (S1 Fig), and were found to be evenly distributed across all observable crossover points.

**Table 6:** PCR-meditated recombination rate by *Taq* DNA polymerase.

Template pair	N_re_ ^a^	N_total_ ^b^	Recombination rate ^c^	Strands with at least 1 recombination event
DNA-1:DNA-1x	19,943	77,725,936	9.6 × 10^−5^	23%
DNA-2:DNA-2x	14,687	44,271,304	1.3 × 10^−4^	28%

^a^ Number of recombination events.

^b^ Total number of analyzed sequenced bases.

^c^ Recombination rate is per base per doubling. Recombination rate is doubled to account for “cryptic” recombination events.

### Plasmid sequencing and thermocycling-induced DNA damage

In addition to polymerase-mediated errors, the level of non-enzymatic base substitution errors resulting from DNA damage during thermocycling was investigated using libraries made from restriction fragments of the plasmid pWB407 [7]. The error rate of bacterial replication was previously measured to be in the range of 10^−9^ to 10^−10^ [26, 27]. In a typical PacBio SMRT sequencing experiment, 10^7^ high-quality consensus bases are obtained per sample, which is at least two orders of magnitude below bacterial replication fidelity, and consequently, plasmid samples are expected to contain near undetectable levels of replication errors. Therefore, errors detected in plasmid samples most likely reflect background sequencing errors or DNA damage introduced during sample preparation. To distinguish between base substitution errors and DNA damage, an enzymatic DNA repair cocktail, PreCR Enzyme Repair Mix, was employed [28]. PreCR is an enzyme blend that recognizes and repairs several types of DNA damage, including mutagenic lesions resulting from cytosine deamination and guanine oxidation.

**Fig 4:**
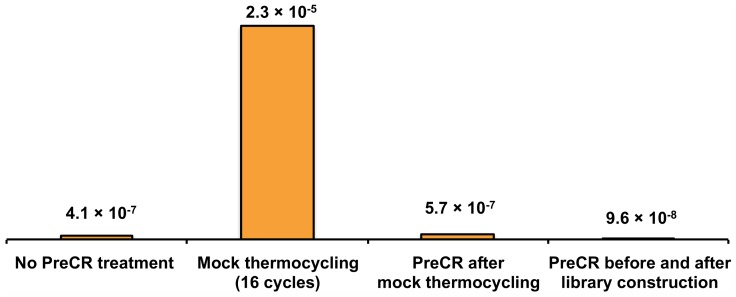
Thermocycling-induced DNA damage. The base substitution error rates of plasmid libraries without treatment, mock thermocycling, and mock thermocycling and PreCR treatment. The base substitution error rate of plasmid libraries treated with PreCR before and after SMRTbell library construction represents the background error rate of the single-molecule sequencing fidelity assay.

To study the effect of thermocycling on DNA, the plasmid samples were mock thermocycled without polymerase present and sequenced before and after temperature cycling. Thermocycling introduced a significant number of mutations, at a rate of 2.3 × 10^−5^ per base over 16 cycles, an increase of 56-fold from before thermocycling (Fig 4). Per PCR cycle, thermocycling-induced damage amounted to 1.4 x 10^−6^ base/cycle. Almost all of the mutations introduced by heating were C→T substitutions (97%), consistent with DNA damage resulting from cytosine deamination (S4 Table). Treating the thermocycled plasmid library with PreCR reduced the mutation rate to pre-heating levels (5.7 × 10^−7^), supporting the identification of C→T substitution errors as the result of cytosine deamination.

Plasmid libraries were also used to measure the background error rate of the single-molecule fidelity assay. For base substitutions, the lowest error rate detected for any plasmid sample was 9.6 × 10^−8^ substitutions/base (Fig 4), achieved by treating the plasmid with PreCR both before and after library construction. For the same sample, deletion and insertion (indel) rates were measured to be 2.4 × 10^−6^ and 7.1 × 10^−7^ errors/base, respectively, indicating that the single-molecule sequencing fidelity assay had a much higher background rate for detecting insertions and deletions and these types of errors could not confidently be reported.

## Discussion

PacBio SMRT sequencing was used to identify and measure different types of errors generated during PCR. The single-molecule sequencing assay was validated by traditional high throughput Sanger sequencing for the canonical *Taq* polymerase. For *Taq* polymerase, which has a total error rate of 1.8 × 10^−4^ errors/base/doubling, single-molecule sequencing was sufficient to detect all types of polymerase errors, including base substitutions, deletions and insertions. For high fidelity polymerases, which can have 1 or 2 orders of magnitude higher fidelity (Table 3), base substitution errors were confidently measured above background error but the accurate detection of rarer deletion and insertion events still remained challenging. For plasmid samples, expected to have undetectable levels of deletions and insertions, the combined insertion and deletion rate was measured to be 3.1 × 10^−6^ indels/base, which represents the background indel rate for the sequencing assay. The background indel error rate was most likely due to remaining homopolymer run-length errors (insertions and deletions) resulting from the inherently high single-pass sequencing indel rate combined with alignment artifacts, and obscured the detection of true indels.

In this study, fidelity measurements generally compare with what was previously reported for *Taq*, Phusion and Q5 DNA polymerases by next-generation sequencing methods. For example, *Taq* polymerase (1.8 × 10^−4^ total errors/base/doubling) was found to have a similar error rate as measured by a primer extension assay (2.0 × 10^−4^ errors/base) [16]. The base substitution rate of Q5 DNA polymerase (5.3 × 10^−7^ sub/base/doubling, in this study) was also measured by a primer extension assay to be 4.0 × 10^−6^ substitutions/base, higher than what was measured in this study, although this number could possibly include errors introduced by DNA damage as well as polymerase errors [16]. In the primer extension fidelity assay by Lee *et al*., one extension product from Q5 polymerase contained abnormally high C→A substitutions and was excluded from analysis [16]. The abnormally high C→A substitution rate was suggested to be a result of guanine oxidation in the template strand. When replicated, the major forms of oxidized guanine pair with adenine, and produce C→A base substitutions [29]. Similarly, in this study, Q5 DNA polymerase was also found to have an extremely low error rate, and produced a higher fraction of C→A substitutions than the other Family B polymerase assayed (Fig 1), suggesting that DNA damage may be a larger contributor to base substitution errors detected for very accurate polymerases than previously thought.

Other fidelity studies involving next-generation sequencing reported error rates per number of PCR cycles instead of per replication doubling event, which can make it challenging to compare measurements from different studies. It is worth emphasizing that replication is not perfectly efficient, which results in a lower number of doubling events than PCR cycles. Furthermore, replication becomes less efficient in later PCR cycles as the reaction exits the log phase [30]. Both factors artificially decrease polymerase error rates, especially if amplification is performed over many PCR cycles. Nonetheless, for comparison to previous studies, the fidelity measurements from this study were converted to normalize per number of PCR cycles (S5 Table). Hestand *et al*. measured the fidelity of *Taq* polymerase to be 4.4 × 10^−5^ errors/base/cycle, and when normalized for the number of PCR cycles, our *Taq* measurements of 5.7 × 10^−5^ sub/base/cycle are similar [15]. Phusion DNA polymerase (1.3 × 10^−6^ sub/base/cycle in this study) was found to have a similar error rate by single-molecule sequencing (1.8 × 10^−6^ sub/base/cycle) [15], but a lower error rate by another Illumina-based method (4.5 × 10^−7^ total errors/base/cycle) [5], indicating there may be some discrepancy in the literature regarding the error rate of Phusion DNA polymerase.

Deep sequencing of a *lacZ* gene fragment revealed how polymerases behave when replicating structured genetic elements. Multiple regions of *lacZ* flanked by inverted repeats showed a propensity to induce polymerase template-switching between top and bottom strands, and were identified by inverted regions in the sequencing read. The polymerase’s replication path was indicated by the sequencing read, but the exact mechanism for how these reads were generated could not be elucidated by sequencing. Two potential mechanisms support the observed template-switching reads: 1) the polymerase switched templates in a single pass, or 2) recombinant reads were generated over multiple polymerase extensions and PCR cycles (S2 Fig). Both mechanisms find support in the literature. Odelberg *et al*. showed that recombination can occur in a single round of extension to support the first mechanism [22]. Alternatively, structured elements can induce polymerase pausing, suggestive of the second mechansim [31]. Further enzymatic studies will reveal whether one or both mechanisms contribute to template-switching.

The prevalence and implication of the template-switch-inducing elements could potentially be greater than what has been revealed in the present study. These experiments only amplified a portion of the *lacZ* gene; more template-switching inducing sequence elements may yet be identified. As the *lacZ* gene is frequently used to measure replication fidelity in blue/white phenotypic assays, specific sequences which cause large sequence rearrangements during amplification may have an effect on the total error rate of the polymerase being measured, particularly as different polymerases have different susceptibilities for introducing errors over these structural elements. For example, Deep Vent and *Pfu* DNA polymerases were previously reported to be 3- and 6-fold higher fidelity than *Taq* polymerase, as measured by a lacZα complementation assay [9]. However, in this study, single-molecule sequencing measured the base substitution rates of Deep Vent and *Pfu* to be 44- and 30-fold higher fidelity than *Taq*, much higher than previously measured. Deep Vent and *Pfu* DNA polymerases were also the most susceptible to template-switching during amplification of *lacZ*. Both enzymes may have a much lower base substitution rate than previously thought, but a higher susceptibility to other types of errors induced by structured elements contained within the *lacZ* gene. Using a single-molecule sequencing-based assay, both error types were identified and their individual error frequencies reported.

By sequencing plasmid DNA, the background error rate for the single-molecule sequencing fidelity assay was 9.6 × 10^−8^ errors/base. The background error rate can also be useful to estimate the lower limits of the single-molecule sequencing fidelity assay. For very accurate polymerases, such as Q5 DNA polymerase, the rate of base substitution errors approaches the background error rate of the assay. For other next-generation sequencing-based fidelity assays, the lower threshold for determining polymerase error rates by barcoded Illumina sequencing was reported as 1 × 10^−6^ errors/base, and attributed to barcode fidelity [32]. Single-molecule sequencing has lowered the threshold for error rate determination, yet as engineered DNA polymerases become more accurate, accurately measuring the error rates of extremely high fidelity polymerases still remains challenging.

As engineered polymerases achieve higher accuracy, other mutagenic sources start to become significant contributors to mutations in PCR products. For example, under Q5 cycling conditions, the amount of mutagenic DNA damage introduced during thermocycling was measured to be 1.4 × 10^−6^ per base per PCR cycle, or once per 714,000 bases, and identified primarily as deoxyuridine resulting from cytosine deamination. This level of damage may be insignificant compared to the error rate of low-fidelity polymerases, such as *Taq* DNA polymerase, but can be a significant contribution to the total errors generated by high fidelity polymerases. For example, Q5 DNA polymerase has an inherent base substitution rate measured to be 5.3 × 10^−7^ sub/base/doubling by single-molecule sequencing, more than two-fold lower than the mutagenic damage introduced by one round of temperature cycling. In the case of Q5 polymerase, DNA damage induced by thermocycling has a much larger contribution to base substitution errors in PCR products than polymerase misincorporation. For certain methods, such as bisulfite sequencing to identify 5-methylcytosine modifications in genomic DNA, the presence of elevated levels of deoxyuridine in PCR products may contribute to false positive methylation sites [33]. Understanding the role that persistent DNA damage plays in contributing to mutations in PCR product will help to identify and avoid false sequence information.

The single-molecule sequencing fidelity assay was further developed to measure the rate of PCR-mediated recombination. In this study, a single-molecule sequencing assay was used to measure the rate and map the location of recombination events in PCR. For *Taq* polymerase, recombination events occurred at an average rate of 1.1 × 10^−4^ per base per doubling event, after normalization for error accumulation in PCR. After 16 cycles of PCR, 23% and 28% of strands contained at least one recombination event, in the range for what has been previously reported for other amplification targets.

The recombination rate is similar to the total misincorporation error rate for *Taq* polymerase, and it remains an open question whether recombination and misincorporation are related. For the artificial amplicons tested, which were designed to be less likely to contain structured or repetitive elements, there was an even distribution of mutation events and little or no sequence specificity for recombination events. Further experiments are necessary to measure recombination events on natural genetic sequences where recombination sites have been identified. For example, for the amplification of the HIV *tat* gene, distinct recombination sites were found, indicating that polymerase pausing may be induced by specific sequence context or structured elements [21]. One hypothesis that has been proposed for the mechanism for inducing recombination is polymerase pausing on specific sequence elements, causing aborted replication and template-switching during the next round of PCR (S2 Fig). Polymerase replication termination could follow polymerase misincorporation or, in the case of archaeal DNA polymerases, uracil-induced replication stalling [34, 35]. Further studies using single-molecule sequencing will elucidate these relationships.

In conclusion, a single-molecule sequencing assay was used to measure the frequency of various types of PCR errors, from single-nucleotide base substitutions to large-scale template-switching events. As PCR errors are propagated during exponential amplification, seemingly infrequent events can have profound implications for downstream analysis, particularly for next-generation sequencing. Single-molecule sequencing can further yield insight into the polymerase behavior responsible for such errors.

## Methods

All reagents are from New England Biolabs, unless otherwise stated.

### Plasmids, template preparation and PreCR treatment

For amplification, 3 different templates were chosen: a 1.4 kb fragment of the *lacZ* gene and two artificial DNA sequences (1.1 kb each). The two artificial DNA sequences, named DNA-1 and DNA-2, were generated randomly *in silico* by combining all possible 4-base combinations, thus ensuring an equimolar base composition. The artificial sequences were also designed to eliminate homopolymer regions greater than 5 bases and include multiple DpnI sites for degradation of methylated plasmid DNA templates after amplification. Two additional artificial DNA sequences, named DNA-1x and DNA-2x, were generated by mutating 9 and 11 marker locations on DNA-1 and DNA-2, respectively (sequences available in the S1 Methods).

The *lacZ* gene is encoded within plasmid pWB407 (6). The artificial sequences DNA-1, DNA-1x, DNA-2 and DNA-2x were synthesized by GenScript and cloned into the EcoRV site of pUC57-Simple or pUC57-mini, respectively. Plasmids were purified from an overnight bacterial culture using a Monarch Plasmid Miniprep kit, then digested with SfoI (pWB407) or XmnI (DNA-1 and DNA-2) in 1X ThermoPol buffer and treated with PreCR (1X ThermoPol buffer, 100 μM each dNTP, 1X NAD^+^, and 1 μl of PreCR Repair mix in a total volume of 50 μl, for 20 minutes at 37°C). Repaired plasmids were cleaned using a Monarch PCR & DNA clean-up kit and the concentration was measured using a Bioanalyzer DNA 12000 kit (Agilent).

### PCR

Each amplification reaction was set up according to the manufacturer’s suggested protocols. Primer and reference sequences, polymerase-specific reaction conditions and cycling protocols are listed in the S1 Methods.

Q5 High Fidelity DNA Polymerase (#M0491), Phusion High Fidelity DNA Polymerase (#M0530), Deep Vent DNA Polymerase (#M0258), and *Taq* DNA polymerase (#M0273) are supplied by New England Biolabs. Other polymerases were purchased from: Cloned *Pfu* DNA Polymerase (Agilent Technologies, #600153), Kapa HiFi HotStart ReadyMix (Kapa Biosystems, #KK2602), KOD DNA Polymerase (Novagen, EMD Millipore # 71085–3), PrimeSTAR GXL DNA Polymerase (Clontech, Takara #R050A).

Reaction conditions ranged from: 1X reaction buffer (provided with the enzyme), 200–300 μM each dNTP, 0.2–1 μM forward and reverse primers, 100 pg/μl plasmid template (linearized with a restriction enzyme and treated with PreCR) and polymerase. Master mix formulations were diluted to 1X final concentration with primers and template. Cycling conditions varied with enzyme according to manufacturer’s recommendation, and all reactions were cycled for 16 cycles. Amplification product yield was determined by measuring the concentration of unpurified PCR products using a Bioanalyzer DNA 12000 kit (Agilent). PCR products were diluted in 10 mM Tris-HCl pH 8.5 (typically requiring between 1/10 to 1/30 dilution) and at least 3 dilutions were averaged.

### Sanger sequencing

PCR was set up as described above with the following primers: *lacZ* forward primer (5’-AAAAACACGAGGTCTCACCTGGCATCGCCTTCTATCGCCTTCTTGACG-3’) and reverse primer (5’-ATATACACGAGGTCTCATTCCGCCGTTCAGCAGCAGCAGACCAT-3’) to amplify a 1 kb fragment of the *lacZ* gene in pWB407 (amplicon reference name, LacZ-1). The primers contain BsaI sites for Golden Gate assembly into the plasmid pSingleD (Rebecca Kucera, New England Biolabs) and BssSI sites for ligating SMRTbell adaptor 5TCGT for PacBio sequencing. Amplification products were cleaned using a Monarch PCR & DNA cleanup column, digested with 20 U BsaI-HF and 20 U DpnI in CutSmart buffer, and cleaned again with a Monarch column. For Golden Gate assembly, the vector fragment was amplified from 4 ng pSingleD using the forward primer (5’-GTAAAGCCTGGGGTGCCTAATGAG-3’) and back primer (5’-TATTTTCTCCTTACGCATCTGTG-3’) and Q5 High Fidelity DNA Polymerase in a total volume of 200 μl. Reactions were cycled: 98°C-30 sec, 20X (98°C-10 sec, 63°C-10 sec, 72°C-1 min 30 sec), 72°C-1 min. The vector amplicon was purified on a Monarch column, digested with BsaI-HF, and cleaned again. 200 ng of each digested insert and vector fragment were ligated together in a 40 μl reaction containing: 1X T4 DNA ligase buffer, 4000 U T4 DNA Ligase, and 10 U BsaI-HF. 2 μl of the ligation reaction was used to transform NEB Turbo Competent *E*. *coli* (#C2984H), and plated on LB/ampicillin. Individual bacterial colonies were picked, grown, DNA prepped and sequenced by Sanger sequencing with forward (5’-GCATCGCCTTCTATCGCCTTCTTGACG-3’) and reverse (5’-CTTCAGCCTCCAGTACAGCGCGG-3’) primers by GeneWiz LLC. To discriminate between Sanger sequencing errors and polymerase errors, both strands of each clone were sequenced, and only bases with confirmed bases on both strands were included for error rate analysis.

### PacBio SMRTbell library construction

100 μl of each PCR was cleaned using a Monarch PCR & DNA Cleanup Kit (5 μg), and digested with BssSαI and DpnI in CutSmart buffer. Digested products were cleaned on a Monarch cleanup column. 400 pmol of Custom SMRTbell adaptors with complementary BssSI overhangs (SMRTbell 5TCGT, 5’P-TCGTATCTCTCTCTTTTCCTCCTCCTCCGTTGTTGTTGTTGAGAGAGAT-3,’ HPLC purification, synthesized by IDT) were ligated to the digested amplification products using the 2X Instant Sticky-end Ligase Master Mix (50 μl total volume for 30 minutes at room temperature). Ligation products were cleaned again with Monarch column. Noncircular products were degraded with 50 U Exonuclease III (*E*. *coli*) and 5 U Exonuclease VII (40 μl total volume, in 1X Standard *Taq* buffer for 1 hour at 37°C). SMRTbell libraries were size selected and cleaned with a 0.6X volume of AMPure PB reagent (Pacific Biosciences), then treated with PreCR (1X ThermoPol buffer, 100 μM each dNTP, 1X NAD^+^, and 1 μl of PreCR Repair mix in a total volume of 50 μl, for 20 minutes at 37°C), and size selected again with a 0.6X volume of AMPure PB reagent (Pacific Biosciences). Libraries were quantified using a Bioanalyzer and a DNA 12000 kit (Agilent). Polymerase binding reactions and MagBeads were prepared using DNA/Polymerase Binding Kit v2 and MagBead Kit v2 (both from Pacific Biosciences) and protocols generated from the Pacific Biosciences’ Binding Calculator (Version 2.3.1.1) [36] and the following custom loading range: 0.0125–0.0225 nM Concentration On Plate to optimize P1 loading density.

### Plasmid sequencing

30 μg of plasmid pWB407 was digested (60 U SspI-HF, 60 U BsaAI in CutSmart buffer in a total volume of 600 μl for 1 hour at 37°C), and digested products were ligated to SMRTbell blunt adaptor (5’P-ATCTCTCTCTTTTCCTCCTCCTCCGTTGTTGTTGTTGAGAGAGAT-3’). Post ligation, reactions were split into thirds and cleaned up on three PCR cleanup-25 columns (Zymo), then processed as described above for PCR products. Mock-heated samples were thermocycled in 1X Q5 Reaction Buffer for 98°C-30 sec, 16 cycles of (98°C-10 sec, 68°C-10 sec, 72°C-30 sec), 72°C-1 min. PreCR treated samples were digested with AfeI and ScaI-HF in CutSmart buffer and ligated to blunt SMRTbell adaptors and then processed as above.

## Building strand-specific consensus sequences

SMRT sequencing was performed on a Pacific Biosciences RSII sequencer with P6 polymerase and C4 chemistry (Pacific Biosciences DNA Sequencing Reagent 4.0 v2) for 6 hour movies. Sequencing data files for each Pacific Biosciences SMRT sample were converted to BAM format using the bax2bam command line tool from SMRT Link v3.1.1 software suite (Pacific Biosciences). Converted subreads were mapped to a corresponding reference sequence for each sample using the BLASR aligner to determine strand directionality. Subreads that mapped to the forward and reverse strand were separated to create two data sets. Consensus sequences were then built separately for each set with the Arrow algorithm using ccs command line tool from SMRT Link software suite, which builds consensus sequence for each individual ZMW from individual subreads. Thus, consensus sequences were built separately for top and bottom strands for each individual ZMW.

### Determining mutational error rates

The resulting high accuracy consensus sequences were mapped to the corresponding reference sequence using the BLASR aligner from the SMRT Link software suite. Base substitutions, deletions and insertions were determined along with corresponding quality values using custom scripts according to Pacific Biosciences recommendations [37]. Since the major factor affecting the quality of consensus sequences is the number of passes (i.e. how many individual subreads were used to build a consensus sequence), at least 15 passes were required for a consensus read to be considered for further analysis. A number of additional filtering criteria were applied before deriving the final error rates to avoid spurious artifacts: (1) the quality values of individual base substitutions, deletions and insertions (QUAL) were required to be the maximum possible value of 93 to ensure highest possible quality of derived consensus bases; (2) the mapping quality value of the aligned read (MAPQ) was required to be 254 (the highest possible) to ensure unambiguous alignments; (3) primer sites were excluded from error calculations; (4) the length of the consensus reads was required to be at least 80% of the expected amplicon length. Additionally, chimeric reads that mapped to more than one region in the reference sequence, as determined by BWA, were discarded.

Only consensus reads and individual base substitutions, deletions and insertions passing the filters above were used to derive error rates. Error rates for polymerase samples were normalized to account for base composition of each amplicon. Raw substitutions error rates were determined separately for A,T ($e_{substitution}^{AT}$) and G,C bases ($e_{substitution}^{GC}$) and resulting substitution error rates (*e*_*substitution*_) were taken as an average, as follows:

$$e_{substitution}^{AT}=\frac{N^{A\to C,A\to G,A\to T}+N^{T\to A,T\to C,T\to G}}{N^{A}+N^{T}},$$ (1)

$$e_{substitution}^{GC}=\frac{N^{G\to A,G\to C,G\to T}+N^{C\to A,C\to G,A\to T}}{N^{G}+N^{C}},$$ (2)

$$e_{substitution}=\frac{e_{substitution}^{AT}+e_{substitution}^{GC}}{2},$$ (3)

where *N*^*A*^, *N*^*T*^, *N*^*G*^, *N*^*C*^ is the total number of analyzed A, T, G, C consensus bases, *N*^*A*→*C*,*A*→*G*,*A*→*T*^, *N*^*T*→*A*,*T*→*C*,*T*→*G*^, *N*^*G*→*A*,*G*→*C*,*G*→*T*^, *N*^*C*→*A*,*C*→*G*,*A*→*T*^ is the number of substitutions involving A, T, G, and C bases respectively. For the plasmid samples, raw substitution error rates were calculated by dividing the number of substitutions by the total number of bases. Deletion and insertions error rates were determined as follows:

$$e_{deletion}=\frac{N_{deletion}}{N},$$ (4)

$$e_{insertion}=\frac{N_{insertion}}{N}.$$ (5)

Lastly, the raw error rates were normalized to account for error propagation in PCR, since errors introduced early in PCR get propagated through subsequent PCR cycles. On average, the fraction of errors *f* in PCR grows linearly with number of doubling events *f* = *n* × *e*/2, where *e* is the polymerase error rate per doubling event, *n* is the number of doubling events [10]. Therefore, the polymerase error rates per doubling event were determined as follows:

$$e^{normalized}=\frac{f\times 2}{n},$$ (6)

where *f* is derived according to Eqs 3–5 for substitutions, deletions and insertions accordingly, and the number of doubling events *n* was found based on the measured product yield after PCR amplification and the input amount of DNA as *n* = log_2_(*Y*/*I*), where *Y* is the yield and *I* is the input amount. The custom scripts used in this study are publicly available at https://github.com/potapovneb/pcr-fidelity.

### Detecting inversions

To detect reads containing inversions, reference sequences corresponding to the correct template and three possible inversions types were constructed (Fig 2). The reference sequences included parts of the sequence corresponding to an inversion region (including both a stem and a loop) and 30 adjacent nucleotides on both sides. Strand-specific consensus reads were derived as described above, with each read requiring at least three passes. The obtained reads were mapped to all four references and the reference sequence with the best match was identified for each read. To ensure high-quality alignments, the aligned part of the read had to cover more than 95% of the reference sequence and no mismatches were allowed. Disallowing mismatches was necessary for the smaller inversion region since an alignment between the correct and double-switch reference sequences contains only two mismatches and two indels. Percentage of reads with inversions is summarized in Table 5 with additional information for each inversion type provided in S3 Table.

### Estimating PCR-mediated recombination rate

To estimate PCR-mediated recombination rates, synthetic templates DNA-1 and DNA-1x, and DNA-2 and DNA-2x, were mixed in an equimolar ratio (10 ng/μl final concentration) and added to a PCR reaction with *Taq* polymerase (1X ThermoPol buffer, 200 μM each dNTP, 0.2 μM forward primer, 0.2 μM reverse primer, 100 pg/μl plasmid template mix, 0.025 U/μl *Taq* polymerase). Reactions were cycled [95°C-30 sec, 16X (95°C-15 sec, 58°C-15 sec, 68°C-1 min 15 sec), 68°C-1 min] and PCR products were processed and sequenced as described above for SMRTbell library construction. Data analysis was performed as follows: strand-specific consensus sequences with at least 3 passes were built and mapped to the reference. Then, the identity of bases at every marker position was determined. To ensure unambiguous alignment around the marker positions, two bases on either side of each marker were required to match the reference sequence. The number of recombination events was then determined in each strand by identifying cases in which marker from one template is followed by markers from the other template (Fig 3). The total number of recombination events was doubled before being divided by the total number of bases to arrive at a raw recombination rate. The raw recombination rate was normalized to account for error propagation in PCR using Eq 6.

## Accession numbers

The raw sequencing data was deposited into the Sequence Read Archive (SRA) with an accession number SRP095133.

## Supporting Information

S1 Fig Distribution of recombination events per interval for Taq DNA polymerase. (PDF)

S2 Fig Possible mechanisms for generating template-switching reads. (PDF)

S1 Methods PCR reaction conditions and cycling protocols for each polymerase, primer and nucleotide sequences. (PDF)

S1 Table DNA Polymerase base substitution rates for individual amplicons. (PDF)

S2 Table Distribution of individual error types for DNA polymerases. (PDF)

S3 Table Percentage of template-switching reads. (PDF)

S4 Table Number of individual error types in plasmid sequencing. (PDF)

S5 Table Polymerase substitution rates normalized by number of PCR cycles. (PDF)
