# Correction: Examining Sources of Error in PCR by Single-Molecule Sequencing

**DOI:** 10.1371/journal.pone.0181128

**Published:** 2017-07-06

**Authors:** Vladimir Potapov, Jennifer L. Ong

In Fig 1A, the labels “PrimeSTAR GXL” and “KOD” are switched. Please see the corrected Fig 1 here.

**Fig 1:**
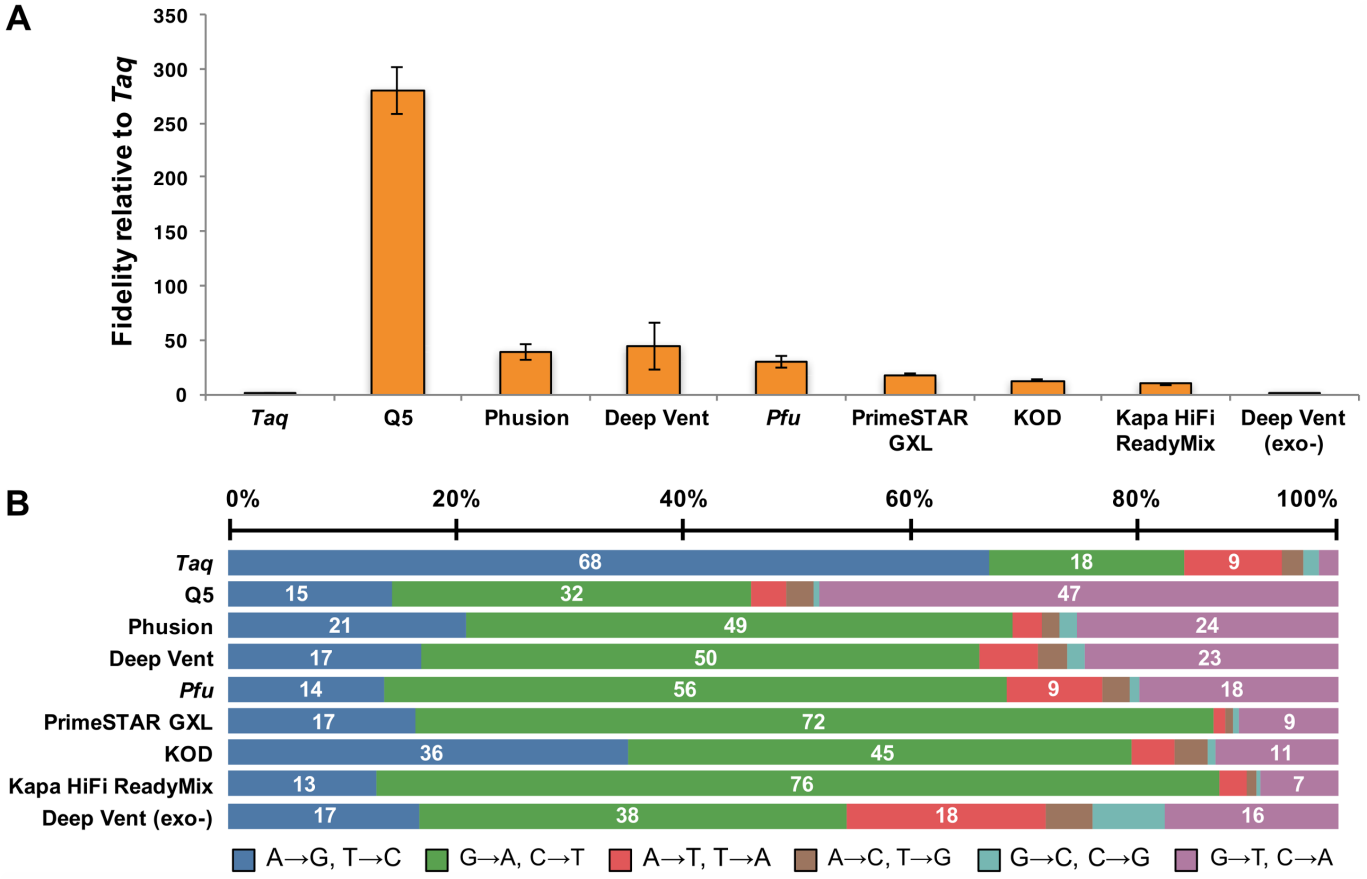
Fidelity measurements and mutational spectrum of DNA polymerases. (A) Base substitution error rates of various DNA polymerases relative to Taq polymerase. (B) Proportion of each type of base substitution error as a percentage of the total errors for each polymerase.
