# Supplementary figures and images for: Examining Sources of Error in PCR by Single-Molecule Sequencing
Source: PLoS One. 2017 Jan 6;12(1):e0169774. doi: 10.1371/journal.pone.0169774 (PMC5218489; doi:10.1371/journal.pone.0169774)

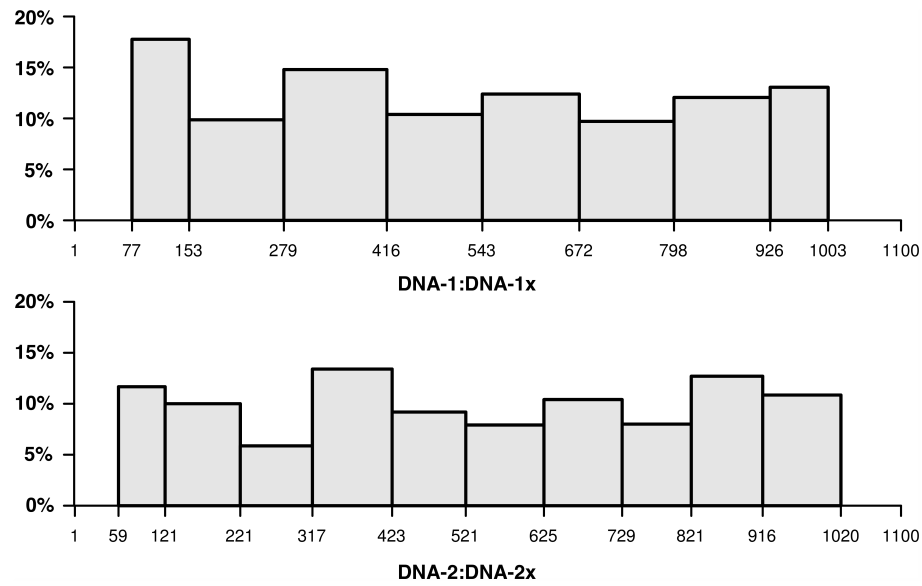

**S1 Fig. Distribution of recombination events per interval for *Taq* DNA polymerase.**

Supplement: S1 Fig — (PDF) [file pone.0169774.s001.pdf]
