# Supplementary material for: Examining Sources of Error in PCR by Single-Molecule Sequencing
Source: PLoS One. 2017 Jan 6;12(1):e0169774. doi: 10.1371/journal.pone.0169774 (PMC5218489; doi:10.1371/journal.pone.0169774)
Supplement: S2 Fig — (PDF) [file pone.0169774.s002.pdf]

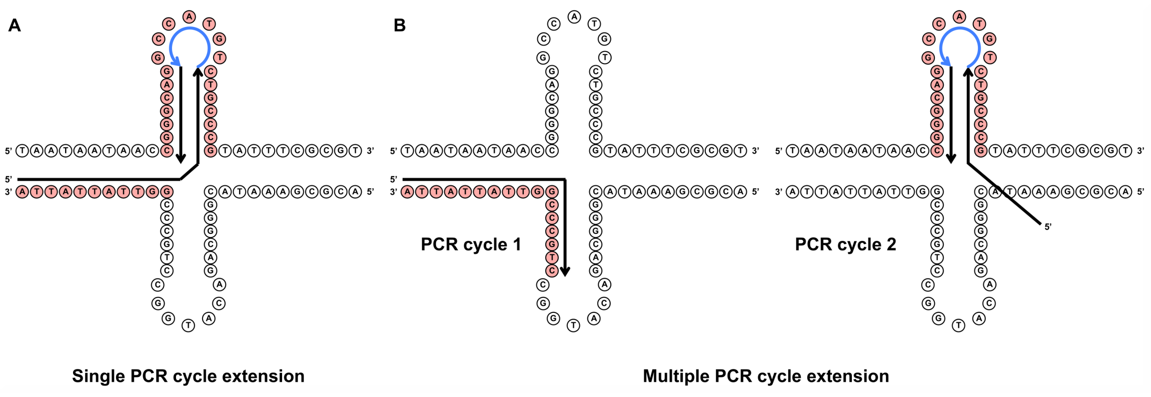

S2 Fig. Possible mechanisms for generating template-switching reads. (A) The polymerase switches templates in a single pass. **(B)** The polymerase terminates after replicating the first half of the inverted repeat. During the next cycle, the extension product anneals to the same repeat on the opposite strand to complete extension.
