# Supplementary material for: Examining Sources of Error in PCR by Single-Molecule Sequencing
Source: PLoS One. 2017 Jan 6;12(1):e0169774. doi: 10.1371/journal.pone.0169774 (PMC5218489; doi:10.1371/journal.pone.0169774)
Supplement: S1 Methods — (PDF) [file pone.0169774.s003.pdf]

# S1 Methods

## PCR reaction conditions and cycling protocols for each polymerase

All reactions were set up for 150 - 200 µl total volume and then split into multiple 50 µl reactions for thermocycling.

***Taq* DNA Polymerase (NEB, catalog # M0267)**

1X ThermoPol buffer
200 µM each dNTP
0.2 µM forward primer
0.2 µM reverse primer
100 pg/µl plasmid template DNA
0.025 U/µl *Taq* DNA polymerase
Cycling protocol:
95°C – 30 sec, 16X (95°C – 15 sec, 58°C – 15 sec, 68°C – 1 min 15 sec), 68°C – 1 min

**Q5 DNA Polymerase (NEB, catalog # M0491)**

1X Q5 Reaction Buffer
200 µM each dNTP
0.5 µM forward primer
0.5 µM reverse primer
100 pg/µl plasmid template DNA
0.02 U/µl Q5 DNA polymerase
Cycling protocol:
98°C – 30 sec, 16X (98°C – 10 sec, 68°C – 10 sec, 72°C – 30 sec), 72°C – 1 min

**Phusion DNA Polymerase (NEB, catalog # M0530)**

1X Phusion HF Buffer
200 µM each dNTP
0.5 µM forward primer
0.5 µM reverse primer
100 pg/µl plasmid template DNA
0.02 U/µl Phusion DNA polymerase
Cycling protocol:
98°C – 30 sec, 16X (98°C – 10 sec, 68°C – 10 sec, 72°C – 40 sec), 72°C – 1 min

**Deep Vent DNA Polymerase (NEB, catalog # M0258) and Deep Vent (exo-) DNA Polymerase (NEB, catalog # M0259)**

1X ThermoPol Buffer
200 µM each dNTP
0.5 µM forward primer
0.5 µM reverse primer
100 pg/µl plasmid template DNA
0.02 U/µl Deep Vent DNA polymerase or Deep Vent (exo-) DNA polymerase
Cycling protocol:

95°C – 2 min, 16X (95°C – 15 sec, 60°C – 15 sec, 72°C – 1 min 15 sec), 72°C – 1 min

***Pfu* DNA Polymerase (Agilent, catalog # 600153)**

1X Cloned *Pfu* Buffer
200 µM each dNTP
0.25 µM forward primer
0.25 µM reverse primer
100 pg/µl plasmid template DNA
0.05 U/µl *Pfu* DNA Polymerase
Cycling protocol:
95°C – 45 sec, 16X (95°C – 45 sec, 58°C – 45 sec, 72°C – 2 min), 72°C – 1 min

**KOD DNA Polymerase (EMD Millipore cat # 71085-3)**

1X Buffer #1 for KOD DNA Polymerase
200 µM each dNTP
1 mM $MgCl_2$
0.4 µM forward primer
0.4 µM reverse primer
100 pg/µl plasmid template DNA
0.02 U/µl KOD DNA Polymerase
Cycling protocol:
16X (95°C – 15 sec, 60°C – 2 sec, 72°C – 20 sec), 72°C – 1 min

**PrimeSTAR GXL Polymerase (TaKaRa, catalog # R050A)**

1X Buffer #1 for KOD DNA Polymerase
200 µM each dNTP
0.3 µM forward primer
0.3 µM reverse primer
100 pg/µl plasmid template DNA
0.025 U/µl PrimeSTAR GXL DNA Polymerase
Cycling protocol:
16X (98°C – 10 sec, 60°C – 15 sec, 68°C – 1 min 10 sec)

**KAPA HiFi HotStart ReadyMix (KAPA, catalog # KM2602)**

1X KAPA HiFi HotStart ReadyMix
0.3 µM forward primer
0.3 µM reverse primer
100 pg/µl plasmid template DNA
Cycling protocol:
95°C – 3 min, 16X (98°C – 20 sec, 60°C – 15 sec, 72°C – 40 sec), 72°C – 1 min

The following primers and templates were used for each amplicon:

| **Amplicon** | **Forward primer (5'-3')** | **Reverse primer (5'-3')** | **Template Plasmid** |
|---|---|---|---|
| LacZ-1 | aaaaacacgaggtctcacctggcatcgccttctatcgccttcttgacg | atatacacgaggtctcattccgccgttcagcagcagcagaccat | pWB407 |
| LacZ-2 | gttaaccgtcacgagcatcatcct | ttaagcacgagcagcggcgtcagcagttgt | pWB407 |
| DNA-1 | agagtacacgagtcaggctacagcatc | tacagttcacgaggaccgtcaaga | DNA-1 (pUC57-Simple) |
| DNA-2 | agagtacacgagtcaggctacagcatc | tacagttcacgaggaccgtcaaga | DNA-2 (pUC57-mini) |
| DNA-1x | agagtacacgagtcaggctacagcatc | tacagttcacgaggaccgtcaaga | DNA-1x (pUC57-mini) |
| DNA-2x | agagtacacgagtcaggctacagcatc | tacagttcacgaggaccgtcaaga | DNA-2x (pUC57-mini) |

## Nucleotide sequences

```
>lacZ
ATGGATCCGATTACGGACTCACTGGCCGTCGTTTTACAACGTCGTGACTGGGAAAACCCT
GGCGTCACCCAACTTAATCGCCTTGCAGCACATCCCCCTTTCGCCAGCTGGCGTAATAGC
GAAGAGGCCCGCACCGATCGCCCTTCCCAACAGTTGCGCAGCCTGAATGGCGAATGGCGC
TTTGCCTGGTTTCCGGCACCAGAAGCGGTGCCGGAAAGCTGGCTGGAGTGCGATCTTCCT
GAGGCCGATACTGTCGTCGTCCCCTCAAACTGGCAGATGCACGGTTACGATGCGCCCATC
TACACCAACGTGACCTATCCCATTACGGTCAATCCGCCGTTTGTTCCCACGGAGAATCCG
ACGGGTTGTTACTCGCTCACATTTAATGTTGATGAAAGCTGGCTACAGGAAGGCCAGACG
CGAATTATTTTTGATGGCGTTAACTCGGCGTTTCATCTGTGGTGCAACGGGCGCTGGGTC
GGTTACGGCCAGGACAGTCGTTTGCCGTCTGAATTTGACCTGAGCGCATTTTTACGCGCC
GGAGAAAACCGCCTCGCGGTGATGGTGCTGCGCTGGAGTGACGGCAGTTATCTGGAAGAT
CAGGATATGTGGCGGATGAGCGGCACTTTCCGTGACGTCTCGTTGCTGCATAAACCGACT
ACACAAATCAGCGATTTCCATGTTGCCACTCGCTTTAATGATGATTTCAGCCGCGCTGTA
CTGGAGGCTGAAGTTCAGATGTGCGGCGAGTTGCGTGACTACCTACGGGTAACAGTTTCT
TTATGGCAGGGTGAAACGCAGGTCGCCAGCGGCACCGCGCCTTTCGGCGGTGAAATTATC
GATGAGCGTGGTGGTTATGCCGATCGCGTCACACTACGTCTGAACGTCGAAAACCCGAAA
CTGTGGAGCGCCGAAATCCCGAATCTCTATCGTGCGGTGGTTGAACTGCACACCGCCGAC
GGCACGCTGATTGAAGCAGAAGCCTGCGATGTCGGTTTCCGCGAGGTGCGGATTGAAAAT
GGTCTGCTGCTGCTGAACGGCAAGCCGTTGCTGATTCGAGGCGTTAACCGTCACGAGCAT
CATCCTCTGCATGGTCAGGTCATGGATGAGCAGACGATGGTGCAGGATATCCTGCTGATG
AAGCAGAACAACTTTAACGCCGTGCGCTGTTCGCATTATCCGAACCATCCGCTGTGGTAC
ACGCCGTGCGACCGCTACGGCCTGTATGTGGTGGATGAAGCCAATATTGAAACCCACGGC
ATGGTGCCAATGAATCGTCTGACCGATGATCCGCGTTGGCTACCGGCGATGAGCGAACGC
GTAACGCGAATGGTGCAGCGCGATCGTAATCACCCGAGTGTGATCATCTGGTCGCTGGGG
AATGAATCAGGCCACGGCGCTAATCACGACGCGCTGTATCGCTGGATCAAATCTGTCGAT
CCTTCCCGCCCGGTGCAGTATGAAGGCGGCGGAGCCGACACCACGGCCACCGATATTATT
TGCCCGATGTACGCGCGCGTGGATGAAGACCAGCCCTTCCCGGCTGTGCCGAAATGGTCC
ATCAAAAAATGGCTTTCGCTACCTGGAGAGACGCGCCCGCTGATCCTTTGCGAATACGCC
CACGCGATGGGTAACAGTCTTGGCGGTTTCGCTAAATACTGGCAGGCGTTTCGTCAGTAT
CCCCGTTTACAGGGCGGCTTCGTCTGGGACTGGGTGGATCAGTCGCTGATTAAATATGAT
GAAAACGGCAACCCGTGGTCGGCTTACGGCGGTGATTTTGGCGATACGCCGAACGATCGC
CAGTTCTGTATGAACGGTCTGGTCTTTGCCGACCGCACGCCGCATCCAGCGCTGACGGAA
GCAAAACACCAGCAGCAGTTTTTCCAGTTCCGTTTATCCGGGCAAACCATCGAAGTGACC
AGCGAATACCTGTTCCGTCATAGCGATAACGAGCTCCTGCACTGGATGGTGGCGCTGGAT
GGTAAGCCGCTGGCAAGCGGTGAAGTGCCTCTGGATGTCGCTCCACAAGGTAAACAGTTG
ATTGAACTGCCTGAACTACCGCAGCCGGAGAGCGCCGGGCAACTCTGGCTCACAGTACGC
GTAGTGCAACCGAACGCGACCGCATGGTCAGAAGCCGGGCACATCAGCGCCTGGCAGCAG
TGGCGTCTGGCGGAAAACCTCAGTGTGACGCTCCCCGCCGCGTCCCACGCCATCCCGCAT
CTGACCACCAGCGAAATGGATTTTTGCATCGAGCTGGGTAATAAGCGTTGGCAATTTAAC
CGCCAGTCAGGCTTTCTTTCACAGATGTGGATTGGCGATAAAAAACAACTGCTGACGCCG
CTGCGCGATCAGTTCACCCGTGCACCGCTGGATAACGACATTGGCGTAAGTGAAGCGACC
CGCATTGACCCTAACGCCTGGGTCGAACGCTGGAAGGCGGCGGGCCATTACCAGGCCGAA
GCAGCGTTGTTGCAGTGCACGGCAGATACACTTGCTGATGCGGTGCTGATTACGACCGCT
CACGCGTGGCAGCATCAGGGGAAAACCTTATTTATCAGCCGGAAAACCTACCGGATTGAT
```

GGTAGTGGTCAAATGGCGATTACCGTTGATGTTGAAGTGGCGAGCGATACACCGCATCCG
GCGCGGATTGGCCTGAACTGCCAGCTGGCGCAGGTAGCAGAGCGGGTAAACTGGCTCGGA
TTAGGGCCGCAAGAAAACTATCCCGACCGCCTTACTGCCGCCTGTTTTGACCGCTGGGAT
CTGCCATTGTCAGACATGTATACCCCGTACGTCTTCCCGAGCGAAAACGGTCTGCGCTGC
GGGACGCGCGAATTGAATTATGGCCCACACCAGTGGCGCGGCGACTTCCAGTTCAACATC
AGCCGCTACAGTCAACAGCAACTGATGGAAACCAGCCATCGCCATCTGCTGCACGCGGAA
GAAGGCACATGGCTGAATATCGACGGTTTCCATATGGGGATTGGTGGCGACGACTCCTGG
AGCCCGTCAGTATCGGCGGAATTCCAGCTGAGCGCCGGTCGCTACCATTACCAGTTGGTC
TGGTGTCAAAAATAATAATAACCGGGCAGGCCATGTCTGCCCGTATTTCGCGT

>LacZ-1
ACGAGGTCTCACCTGGCATCGCCTTCTATCGCCTTCTTGACGAGTTCTTCTGAAGCTCAG
ATCTCAGGAAACAGCTATGGATCCGATTACGGACTCACTGGCCGTCGTTTTACAACGTCG
TGACTGGGAAAACCCTGGCGTCACCCAACTTAATCGCCTTGCAGCACATCCCCCTTTCGC
CAGCTGGCGTAATAGCGAAGAGGCCCGCACCGATCGCCCTTCCCAACAGTTGCGCAGCCT
GAATGGCGAATGGCGCTTTGCCTGGTTTCCGGCACCAGAAGCGGTGCCGGAAAGCTGGCT
GGAGTGCGATCTTCCTGAGGCCGATACTGTCGTCGTCCCCTCAAACTGGCAGATGCACGG
TTACGATGCGCCCATCTACACCAACGTGACCTATCCCATTACGGTCAATCCGCCGTTTGT
TCCCACGGAGAATCCGACGGGTTGTTACTCGCTCACATTTAATGTTGATGAAAGCTGGCT
ACAGGAAGGCCAGACGCGAATTATTTTTGATGGCGTTAACTCGGCGTTTCATCTGTGGTG
CAACGGGCGCTGGGTCGGTTACGGCCAGGACAGTCGTTTGCCGTCTGAATTTGACCTGAG
CGCATTTTTACGCGCCGGAGAAAACCGCCTCGCGGTGATGGTGCTGCGCTGGAGTGACGG
CAGTTATCTGGAAGATCAGGATATGTGGCGGATGAGCGGCACTTTCCGTGACGTCTCGTT
GCTGCATAAACCGACTACACAAATCAGCGATTTCCATGTTGCCACTCGCTTTAATGATGA
TTTCAGCCGCGCTGTACTGGAGGCTGAAGTTCAGATGTGCGGCGAGTTGCGTGACTACCT
ACGGGTAACAGTTTCTTTATGGCAGGGTGAAACGCAGGTCGCCAGCGGCACCGCGCCTTT
CGGCGGTGAAATTATCGATGAGCGTGGTGGTTATGCCGATCGCGTCACACTACGTCTGAA
CGTCGAAAACCCGAAACTGTGGAGCGCCGAAATCCCGAATCTCTATCGTGCGGTGGTTGA
ACTGCACACCGCCGACGGCACGCTGATTGAAGCAGAAGCCTGCGATGTCGGTTTCCGCGA
GGTGCGGATTGAAAATGGTCTGCTGCTGCTGAACGGCGGAATGAGACCTCGT

>LacZ-2
ACGAGTTCTGTATGAACGGTCTGGTCTTTGCCGACCGCACGCCGCATCCAGCGCTGACGG
AAGCAAAACACCAGCAGCAGTTTTTCCAGTTCCGTTTATCCGGGCAAACCATCGAAGTGA
CCAGCGAATACCTGTTCCGTCATAGCGATAACGAGCTCCTGCACTGGATGGTGGCGCTGG
ATGGTAAGCCGCTGGCAAGCGGTGAAGTGCCTCTGGATGTCGCTCCACAAGGTAAACAGT
TGATTGAACTGCCTGAACTACCGCAGCCGGAGAGCGCCGGGCAACTCTGGCTCACAGTAC
GCGTAGTGCAACCGAACGCGACCGCATGGTCAGAAGCCGGGCACATCAGCGCCTGGCAGC
AGTGGCGTCTGGCGGAAAACCTCAGTGTGACGCTCCCCGCCGCGTCCCACGCCATCCCGC
ATCTGACCACCAGCGAAATGGATTTTTGCATCGAGCTGGGTAATAAGCGTTGGCAATTTA
ACCGCCAGTCAGGCTTTCTTTCACAGATGTGGATTGGCGATAAAAAACAACTGCTGACGC
CGCTGCGCGATCAGTTCACCCGTGCACCGCTGGATAACGACATTGGCGTAAGTGAAGCGA
CCCGCATTGACCCTAACGCCTGGGTCGAACGCTGGAAGGCGGCGGGCCATTACCAGGCCG
AAGCAGCGTTGTTGCAGTGCACGGCAGATACACTTGCTGATGCGGTGCTGATTACGACCG
CTCACGCGTGGCAGCATCAGGGGAAAACCTTATTTATCAGCCGGAAAACCTACCGGATTG
ATGGTAGTGGTCAAATGGCGATTACCGTTGATGTTGAAGTGGCGAGCGATACACCGCATC
CGGCGCGGATTGGCCTGAACTGCCAGCTGGCGCAGGTAGCAGAGCGGGTAAACTGGCTCG

GATTAGGGCCGCAAGAAAACTATCCCGACCGCCTTACTGCCGCCTGTTTTGACCGCTGGG
ATCTGCCATTGTCAGACATGTATACCCCGTACGTCTTCCCGAGCGAAAACGGTCTGCGCT
GCGGGACGCGCGAATTGAATTATGGCCCACACCAGTGGCGCGGCGACTTCCAGTTCAACA
TCAGCCGCTACAGTCAACAGCAACTGATGGAAACCAGCCATCGCCATCTGCTGCACGCGG
AAGAAGGCACATGGCTGAATATCGACGGTTTCCATATGGGGATTGGTGGCGACGACTCCT
GGAGCCCGTCAGTATCGGCGGAATTCCAGCTGAGCGCCGGTCGCTACCATTACCAGTTGG
TCTGGTGTCAAAAATAATAATAACCGGGCAGGCCATGTCTGCCCGTATTTCGCGTAAGGA
AATCCATAATTCTTGAAGACGAAAGGGCCTCGT

>DNA-1
ACGAGTCAGGCTACAGCATCCTCTGGTTCAGACTACTTGATTCATGTGTACCCTATATGC
GAGGATATGTGTATCGTAGAAATTGTCAGGCAGTAACGTTCCGCGAGTTTTAATGGGCGC
GCCATGACTCTAAGAGTGATATACCTCCTCGGTCTCGGGCCCGGGGTGTAATTAGCCCAG
TTAGACACGATCGCCCGACGTATATTGTTGCTTGGGTATCGTCGCATGCGAAGTATTGCC
CAAGGAGACACAACAAGCAACTTATGTTGACTCCCTTCGACCATTAAAATTTGTTAGAAC
GGACAGAAAGGATGCGCCTTATAAATGTCCTGTGCAGTGATGAAGCGACCTCAAAACGCT
TCATGATCTAACCGACTCACCTTGCCGTTCCCTCCGCGCCTTAAAACCGGCCGGTCTTGC
GAAAAGCGGGAAACGAGTTTACCCACGGATAGCAGGGAATGTTGCGGCTGGCTAGGGAGC
ATGAAGGTAGATACTCCACGGCTTACCTTTCCGGGGCTCAACATCTAGCCACAGACCTTT
TCGTTAAGCCCACCCCCACTGGATACTGAATCATCAGGGAACCGGACCCAACCAGTTTGG
GCTCGTCCAAGCTTCGGTCTCGTCCCTAAGTGCAAAGATATGGAAAGAGCAGCATAGGTA
TATGGATTATTCTTTTACCACTCGTTTCTTACCGTAACTTACGCAATGGATCACGTGCCG
AGGCGGCGGTACAGCTGTTCGAAGGGCTCTGTCGGGAACGCTAACATCCAGCCGGTAAAT
TCCAAACTAGGGAAAGGACACGCACTGAATTGAATATAGTCGTGAAGGGTGGTGTAAGTC
GTGCACAGCCCGCATTAAGTACTAAACAGCGTCCAATCTTGATCTACTTACGGCCTGATG
TTCTTCAGCACCTCCTAGCACTGGAGTACTTCGCTATCAATGAGATTAGCACTTTGTACA
TGTCATCCAGCCCGAGTCTGGGGTCCGACAATGCGGTCGCCGATTGGTATCTGCATGTAG
TATTAAACGGAGCTGCCGCGGCTGCGGATTATAGTTCATGTCTTGACGGTCCTCGT

>DNA-2
ACGAGTCAGGCTACAGCATCTTGACACCAGAATATTATGGATTGGACGCTTCCCACTAAA
TGGAAGACTGTTCGGTCATAAACACTACTAGGAATTCCTCTCCAGTCATCATGTTCGATC
GTCTAGCAGCAATCTCTTCCGATCGATATTTGCGCGTGACTCAGGCGAGCCCATGACAGC
TTCTCCCCGTGAGAACCACGACTAGAAGTTATCTGTTGAGCTGCTAGCTTCGTGGCCCGG
CCATGGTAGTAGCGGCTCACTCGCGCTAACTTTGCCTGCTCGAGAAAACGGGCGAAACAC
CCAGCAACACAAGCCACTTAATTTGTTGATAGATAATAAGATCAGGTTATTAGTCGCTCT
GCACTTACTTTAAGTGCCAACTATGCTGTATCGGCCAGGGTGAAAACGGGTGCCGCCACT
TCAGTGTGTCGGAGTCTGCTGACGGATTAGGGCACAGACGTATGGTTATATCCTAAGGTA
GTGTGTCAATGTACTGGGGACAAAGTCAGTGGGCACCGCATCAGGAGTGCAACCTCCGCT
AGTACCGACTCGTCAATGCTTTGAGCGATGGCTTGCGCTCCCAAATCCTTAAGCTTTTAT
GCATTCGGCTCTGGCCCTCAGGCCTGACCTGGAATTTCATCGGAAACGCCTTAACCGACA
TTACATCGACACCAAGATCCCGACGCTTCATGCGGAGACGATAGAGACTCTAACCAAGAA
TAAAAGGAGTAGTCCCTAATCTACTGAAACGGGGATACCTCAAATCACGGGAATGCGTTA
CTGACCCGCTATGTGAGGCTCGGATCACCCTCGTTCTATTGCCTTGTAATCATGGTGGGG
CGGCGGAGCGGGATTAGAGGGTGTCCCTAATGTGAGTAGATCTGTAGTAATGATACGTCT
CCTCAATATGAGGCGTATTGCAGGTCACAGCACAGGGAGATTTCGGCGCACCCAGCCGAG
TTGCCTCCGTCGTTGTTTAGGTATATGCATAACTGCTCACGACAAATACAGCAGAGCCTA

CGTTGGGTTATCGAATCCTTGTGGACAAGAAGCTTCTTCATGTCTTGACGGTCCTCGT

>DNA-1x
ACGAGTCAGGCTACAGCATCCTCTGGTTCAGACTACTTGATTCATGTGTACCCTATATGC
GAGGATATGTGTATCGCAGAAATTGTCAGGCAGTAACGTTCCGCGAGTTTTAATGGGCGC
GCCATGACTCTAAGAGTGATATACCTCCTCGGACTCGGGCCCGGGGTGTAATTAGCCCAG
TTAGACACGATCGCCCGACGTATATTGTTGCTTGGGTATCGTCGCATGCGAAGTATTGCC
CAAGGAGACACAACAAGCAACTTATGTTGACTCCCTTCTACCATTAAAATTTGTTAGAAC
GGACAGAAAGGATGCGCCTTATAAATGTCCTGTGCAGTGATGAAGCGACCTCAAAACGCT
TCATGATCTAACCGACTCACCTTGCCGTTCCCTCCGCGCCTTAAAACCGGCCGGTATTGC
GAAAAGCGGGAAACGAGTTTACCCACGGATAGCAGGGAATGTTGCGGCTGGCTAGGGAGC
ATGAAGGTAGATACTCCACGGCTTACCTTTCCGGGGCTCAACATCTAGCCACAGACCTTT
TCATTAAGCCCACCCCCACTGGATACTGAATCATCAGGGAACCGGACCCAACCAGTTTGG
GCTCGTCCAAGCTTCGGTCTCGTCCCTAAGTGCAAAGATATGGAAAGAGCAGCATAGGTA
TATGGATTATTGTTTTACCACTCGTTTCTTACCGTAACTTACGCAATGGATCACGTGCCG
AGGCGGCGGTACAGCTGTTCGAAGGGCTCTGTCGGGAACGCTAACATCCAGCCGGTAAAT
TCCAAACTAGGGAAAGGTCACGCACTGAATTGAATATAGTCGTGAAGGGTGGTGTAAGTC
GTGCACAGCCCGCATTAAGTACTAAACAGCGTCCAATCTTGATCTACTTACGGCCTGATG
TTCTTCAGCACCTCCTAGCACTGGACTACTTCGCTATCAATGAGATTAGCACTTTGTACA
TGTCATCCAGCCCGAGTCTGGGGTCCGACAATGCGGTCGCCGCTTGGTATCTGCATGTAG
TATTAAACGGAGCTGCCGCGGCTGCGGATTATAGTTCATGTCTTGACGGTCCTCGT

>DNA-2x
ACGAGTCAGGCTACAGCATCTTGACACCAGAATATTATGGATTGGACGCTTCCCACTACA
TGGAAGACTGTTCGGTCATAAACACTACTAGGAATTCCTCTCCAGTCATCATGTTCGATC
ATCTAGCAGCAATCTCTTCCGATCGATATTTGCGCGTGACTCAGGCGAGCCCATGACAGC
TTCTCCCCGTGAGAACCACGACTAGAAGTTATCTGTTGAGATGCTAGCTTCGTGGCCCGG
CCATGGTAGTAGCGGCTCACTCGCGCTAACTTTGCCTGCTCGAGAAAACGGGCGAAACAC
CCAGCAACACAAGCCAGTTAATTTGTTGATAGATAATAAGATCAGGTTATTAGTCGCTCT
GCACTTACTTTAAGTGCCAACTATGCTGTATCGGCCAGGGTGAAAACGGGTGCCGCCACT
TCTGTGTGTCGGAGTCTGCTGACGGATTAGGGCACAGACGTATGGTTATATCCTAAGGTA
GTGTGTCAATGTACTGGGGACAAAGTCAGTGGGCACCGCAGCAGGAGTGCAACCTCCGCT
AGTACCGACTCGTCAATGCTTTGAGCGATGGCTTGCGCTCCCAAATCCTTAAGCTTTTAT
GCATTCGGCTCTGGCCCTCAGGCCAGACCTGGAATTTCATCGGAAACGCCTTAACCGACA
TTACATCGACACCAAGATCCCGACGCTTCATGCGGAGACGATAGAGACTCTAACCAAGAA
TAAAAGGACTAGTCCCTAATCTACTGAAACGGGGATACCTCAAATCACGGGAATGCGTTA
CTGACCCGCTATGTGAGGCTCGGATCACCCTCGTTCTATTACCTTGTAATCATGGTGGGG
CGGCGGAGCGGGATTAGAGGGTGTCCCTAATGTGAGTAGATCTGTAGTAATGATACGTCT
CCTCAATATGAGGCGCATTGCAGGTCACAGCACAGGGAGATTTCGGCGCACCCAGCCGAG
TTGCCTCCGTCGTTGTTTAGGTATATGCATAACTGCTCACGACAAATACAGCAGAGCCTG
CGTTGGGTTATCGAATCCTTGTGGACAAGAAGCTTCTTCATGTCTTGACGGTCCTCGT
