# Supplementary material for: Examining Sources of Error in PCR by Single-Molecule Sequencing
Source: PLoS One. 2017 Jan 6;12(1):e0169774. doi: 10.1371/journal.pone.0169774 (PMC5218489; doi:10.1371/journal.pone.0169774)
Supplement: S1 Table — (PDF) [file pone.0169774.s004.pdf]

S1 Table. DNA Polymerase base substitution rates for individual amplicons

DNA Polymerase / Amplicon	Substitution rate (sub/base/doubling)	Fidelity, rel. to *Taq*	Doubling events	Total bases
Taq				
LacZ-1	1.7E-4	1	11.8	35,879,784
LacZ-2	1.7E-4	1	11.3	15,857,446
DNA-1	1.4E-4	1	12.2	18,680,811
DNA-2	1.4E-4	1	11.8	27,978,748
**Summary**	1.5E-4	1	11.8	98,396,789
**Q5**				
LacZ-2	6.3E-7	271	11.3	58,804,553
DNA-1	5.2E-7	265	11.5	24,031,390
DNA-2	4.5E-7	304	11.3	29,783,285
**Summary**	5.3E-7	280	11.3	112,619,228
**Phusion**				
LacZ-2	4.2E-6	41	10.8	27,516,808
DNA-1	3.1E-6	44	10.6	43,337,948
DNA-2	4.5E-6	30	10.1	47,408,183
**Summary**	3.9E-6	39	10.5	118,262,939
**Deep Vent**				
LacZ-2	5.9E-6	29	10.3	24,256,327
DNA-1	2.0E-6	68	10.6	41,951,269
DNA-2	4.0E-6	34	10.2	40,010,344
**Summary**	4.0E-6	44	10.4	106,217,940
Pfu				
LacZ-2	6.1E-6	28	8.4	31,354,026
DNA-1	4.0E-6	35	8.8	37,838,462
DNA-2	5.3E-6	26	8.8	10,422,488
**Summary**	5.1E-6	30	8.7	79,614,976
**PrimeSTAR GXL**				
LacZ-2	9.5E-6	18	11.5	36,639,913
DNA-1	7.3E-6	19	11.1	40,281,638
DNA-2	8.5E-6	16	11.0	42,043,015
**Summary**	8.4E-6	18	11.2	118,964,566
**KOD**				
LacZ-2	1.4E-5	12	12.0	37,137,931
DNA-1	1.0E-5	13	11.8	39,290,128
DNA-2	1.2E-5	12	11.5	44,806,379
**Summary**	1.2E-5	12	11.8	121,234,438
**Kapa HiFi HotStart ReadyMix**				
LacZ-2	1.9E-5	9	11.9	25,543,632
DNA-1	1.4E-5	10	12.6	33,023,626
DNA-2	1.5E-5	9	12.4	43,175,705
**Summary**	1.6E-5	9	12.3	101,742,963
**Deep Vent (exo-)**				
LacZ-2	5.0E-4	0.3	9.5	23,213,109
DNA-1	5.0E-4	0.3	9.7	13,305,219
DNA-2	4.8E-4	0.3	9.2	23,700,277
**Summary**	5.0E-4	0.3	9.5	60,218,605
