# Supplementary material for: Examining Sources of Error in PCR by Single-Molecule Sequencing
Source: PLoS One. 2017 Jan 6;12(1):e0169774. doi: 10.1371/journal.pone.0169774 (PMC5218489; doi:10.1371/journal.pone.0169774)
Supplement: S2 Table — (PDF) [file pone.0169774.s005.pdf]

S2 Table. Distribution of individual error types for DNA polymerases

DNA Polymerase	A→G, T→C (%)	G→A, C→T (%)	A→T, T→A (%)	A→C, T→G (%)	G→C, C→G (%)	G→T, C→A (%)
Taq	68	18	8.8	2.1	1.3	1.8
Q5	15	32	3.2	2.4	0.5	47
Phusion	21	49	2.7	1.5	1.6	24
Deep Vent	17	50	5.2	2.6	1.7	23
Pfu	14	56	8.6	2.5	0.8	18
PrimeSTAR GXL	17	72	1.0	0.8	0.5	9.0
KOD	36	45	3.9	2.9	0.8	11
Kapa HiFi HotStart ReadyMix	13	76	2.5	1.0	0.3	7.0
Deep Vent (exo-)	17	38	18	4.1	6.6	16
