# Supplementary material for: Examining Sources of Error in PCR by Single-Molecule Sequencing
Source: PLoS One. 2017 Jan 6;12(1):e0169774. doi: 10.1371/journal.pone.0169774 (PMC5218489; doi:10.1371/journal.pone.0169774)
Supplement: S3 Table — (PDF) [file pone.0169774.s006.pdf]

**S3 Table.** Percentage of template-switching reads.

| Enzyme | Double | Single (fwd) | Single (rev) | Total reads |
|---|---|---|---|---|
| ***lacZ inversion region 1843..1981*** | | | | |
| Taq | n.d. | n.d. [a] | n.d. | 29,047 |
| Q5 | n.d. | n.d. [a] | 0.003% | 104,812 |
| Phusion | n.d. | n.d. [a] | n.d. | 73,085 |
| Deep Vent | 0.184% | 0.006% [a] | 1.350% | 67,473 |
| Pfu | n.d. | 0.001% [a] | 0.421% | 67,656 |
| PrimeSTAR GXL | n.d. | n.d. [a] | n.d. | 73,847 |
| KOD | 0.011% | n.d. [a] | n.d. | 55,712 |
| Kapa HiFi HotStart ReadyMix | n.d. | n.d. [a] | 0.006% | 36,229 |
| Deep Vent (exo-) | n.d. | n.d. [a] | 0.028% | 31,644 |
| ***lacZ inversion region 3083..3103*** | | | | |
| Taq | n.d. | n.d. | n.d. [b] | 31,566 |
| Q5 | 0.001% | n.d. | n.d. [b] | 104,340 |
| Phusion | 0.024% | n.d. | n.d. [b] | 71,609 |
| Deep Vent | 0.163% | 0.004% | n.d. [b] | 66,684 |
| Pfu | 0.003% | n.d. | n.d. [b] | 67,492 |
| PrimeSTAR GXL | 0.004% | n.d. | n.d. [b] | 73,653 |
| KOD | 0.400% | 0.002% | n.d. [b] | 55,464 |
| Kapa HiFi HotStart ReadyMix | 0.003% | n.d. | n.d. [b] | 36,185 |
| Deep Vent (exo-) | 0.010% | n.d. | n.d. [b] | 40,651 |

[a] The expected length of template switching read is 227 nt, which is not expected to be detected because its size is below the threshold for sequencing by MagBead loading on the PacBio RSII.

[b] The expected length of template switching read is 117 nt, which is not expected to be detected because its size is below the threshold for sequencing by MagBead loading on the PacBio RSII.
