# Supplementary material for: Examining Sources of Error in PCR by Single-Molecule Sequencing
Source: PLoS One. 2017 Jan 6;12(1):e0169774. doi: 10.1371/journal.pone.0169774 (PMC5218489; doi:10.1371/journal.pone.0169774)
Supplement: S4 Table — (PDF) [file pone.0169774.s007.pdf]

S4 Table. Number of individual error types in plasmid sequencing

Plasmid sample	Substitution rate	A→C	A→T	A→G	C→A	C→T	C→G	T→A	T→C	T→G	G→A	G→C	G→T	N_{sub} [a]	N_{total} [b]
No PreCR treatment	4.1×10^{-7}	0	1	0	0	9	0	0	0	3	3	1	1	18	43,877,869
PreCR before and after library construction	9.6×10^{-8}	0	0	1	0	0	0	0	1	0	0	0	1	3	31,198,374
Mock thermocycling (16 cycles)	2.3×10^{-5}	1	0	25	0	1,471	0	0	2	14	1	0	1	1,515	66,916,627
PreCR after mock thermocycling	5.7×10^{-7}	0	1	5	1	10	0	0	1	0	0	1	1	20	35,278,125

[a] Total number of substitutions.

[b] Total number of analyzed sequenced bases.
