# Supplementary material for: Examining Sources of Error in PCR by Single-Molecule Sequencing
Source: PLoS One. 2017 Jan 6;12(1):e0169774. doi: 10.1371/journal.pone.0169774 (PMC5218489; doi:10.1371/journal.pone.0169774)
Supplement: S5 Table — (PDF) [file pone.0169774.s008.pdf]

S5 Table. Polymerase substitution rates normalized by number of PCR cycles

**DNA Polymerase**	**Substitution rate (sub/base/PCR cycle)**
Taq	5.7×10^{-5} ($\pm$ 0.7×10^{-5})
Q5	1.9×10^{-7} ($\pm$ 0.3×10^{-7})
Phusion	1.3×10^{-6} ($\pm$ 0.2×10^{-6})
Deep Vent	1.3×10^{-6} ($\pm$ 0.6×10^{-6})
Pfu	1.4×10^{-6} ($\pm$ 0.3×10^{-6})
PrimeSTAR GXL	3.0×10^{-6} ($\pm$ 0.4×10^{-6})
KOD	4.5×10^{-6} ($\pm$ 0.8×10^{-6})
Kapa HiFi HotStart ReadyMix	6.0×10^{-6} ($\pm$ 0.8×10^{-6})
Deep Vent (exo-)	1.5×10^{-4} ($\pm$ 0.01×10^{-4})
